# Scale adaptive compressive tracking

**DOI:** 10.1186/s40064-016-2350-y

**Published:** 2016-06-23

**Authors:** Pengpeng Zhao, Shaohui Cui, Min Gao, Dan Fang

**Affiliations:** Electronic Engineering Department, Shijiazhuang Mechanical Engineering College, No. 97 Heping West Road, Shijiazhuang, 050003 China

**Keywords:** Visual tracking, Compressive tracking, Feature template, Model update

## Abstract

Recently, the compressive tracking (CT) method (Zhang et al. in Proceedings of European conference on computer vision, pp 864–877, 2012) has attracted much attention due to its high efficiency, but it cannot well deal with the scale changing objects due to its constant tracking box. To address this issue, in this paper we propose a scale adaptive CT approach, which adaptively adjusts the scale of tracking box with the size variation of the objects. Our method significantly improves CT in three aspects: Firstly, the scale of tracking box is adaptively adjusted according to the size of the objects. Secondly, in the CT method, all the compressive features are supposed independent and equal contribution to the classifier. Actually, different compressive features have different confidence coefficients. In our proposed method, the confidence coefficients of features are computed and used to achieve different contribution to the classifier. Finally, in the CT method, the learning parameter λ is constant, which will result in large tracking drift on the occasion of object occlusion or large scale appearance variation. In our proposed method, a variable learning parameter λ is adopted, which can be adjusted according to the object appearance variation rate. Extensive experiments on the CVPR2013 tracking benchmark demonstrate the superior performance of the proposed method compared to state-of-the-art tracking algorithms.

## Background

Visual object tracking is a significant research hotspot in computer vision because of its numerous applications such as intelligent monitor system, precision guide system, intelligent medical diagnosis, etc. However, it remains a challenging task to develop robust tracking algorithms due to the appearance change caused by illumination, motion, occlusion, and so on. Aimed at this issue, numerous algorithms have been proposed, which can be divided into algorithms based on generative appearance models (Babu et al. 2007; Lim et al. 2004; Adam et al. 2006; Mei and Ling 2009, 2011; Li et al. 2011; Liu et al. 2013) and algorithms based on discriminative appearance models (Avidan 2004; Grabner and Bischof 2006; Grabner et al. 2008; Babenko et al. 2011; Zhang et al. 2013, 2014a, b; Liu et al. 2015).

Generative algorithms typically learn a representative object model, which is utilized to search for the most similar region in image according to a certain similarity principle. Babu et al. (2007) make use of a linear subspace model to represent object appearance for tracking. Lim et al. (2004) utilizes an incremental learning method to update both subspace model and samples average. Adam et al. (2006) use the intensity histograms of multiple fragments to represent object appearance, which can be computed by integral images efficiently. Recently, Mei and Ling (2009, 2011) proposed a robust object tracking method based on the sparse representation theory, named *l*_1_ tracker, which introduces the sparse representation theory into object tracking at the first time. Li et al. (2011) further improved the *l*_1_ tracker by using the orthogonal matching pursuit algorithm for solving the optimization problems efficiently. Liu et al. (2013) propose robust visual tracking method using local sparse appearance model and k-selection, which introduces block cording coefficient into mean shift to search for the optimal tracking result. Despite much progress has been achieved, there are still several problems to be solve in generative tracking algorithms. First, numerous training samples are required for learning an object appearance model at the start. However, if the object appearance change significantly during this period, the drift problem is likely to occur. Secondly, all of these generative algorithms don’t use the background information, which is likely helpful to improve the tracking results.

Discriminative algorithms regard object tracking as a binary classification task, the goal of which is to find the optimal classify function between different classes. Avidan (2004) makes use of an off-line support vector machine (SVM) classifier to design a tracker. Grabner and Bischof (2006) propose an on-line features selected visual tracking method by using Adboost algorithm to select features on line. Soon afterwards, Grabner et al. (2008) propose semi-supervised on-line boosting for robust tracking, and the key of the method combines the advantage of both on-line and off-line classifier. Babenko et al. (2011) treat the tracking task as a multiple instance learning (MIL) problem, and propose a robust object tracking method with online MIL. Zhang et al. (2013) point out the shortcoming of on-line MIL, and propose a new tracking method, named ODFS, by introducing features selection into on-line MIL system. Recently, Zhang et al. (2012) propose a real-time compressive tracking (CT) algorithm that employs a very sparse random matrix to achieve a low-dimensional object appearance representation. Soon afterwards, Zhang et al. (2014a, b) further improve CT algorithm by reducing computational complexity. Liu et al. (2015) point out the shortness of CT algorithm, and propose adaptive compressive tracking method via online vector boosting feature selection.

In this paper, we propose a scale adaptive CT method which can adaptively adjusts the scale of tracking box with the size variation of the objects. Furthermore, the confidence coefficients of features are computed and used to achieve different contribution to the classifier. Finally, a variable learning parameter λ is adopted, which can be adjusted according to the object appearance variation rate. Extensive experiments on the CVPR2013 tracking benchmark demonstrate the superior performance of the proposed method compared to state-of-the-art tracking algorithms in terms of efficiency, accuracy and robustness.

## Compressive tracking

**Fig. 1:**
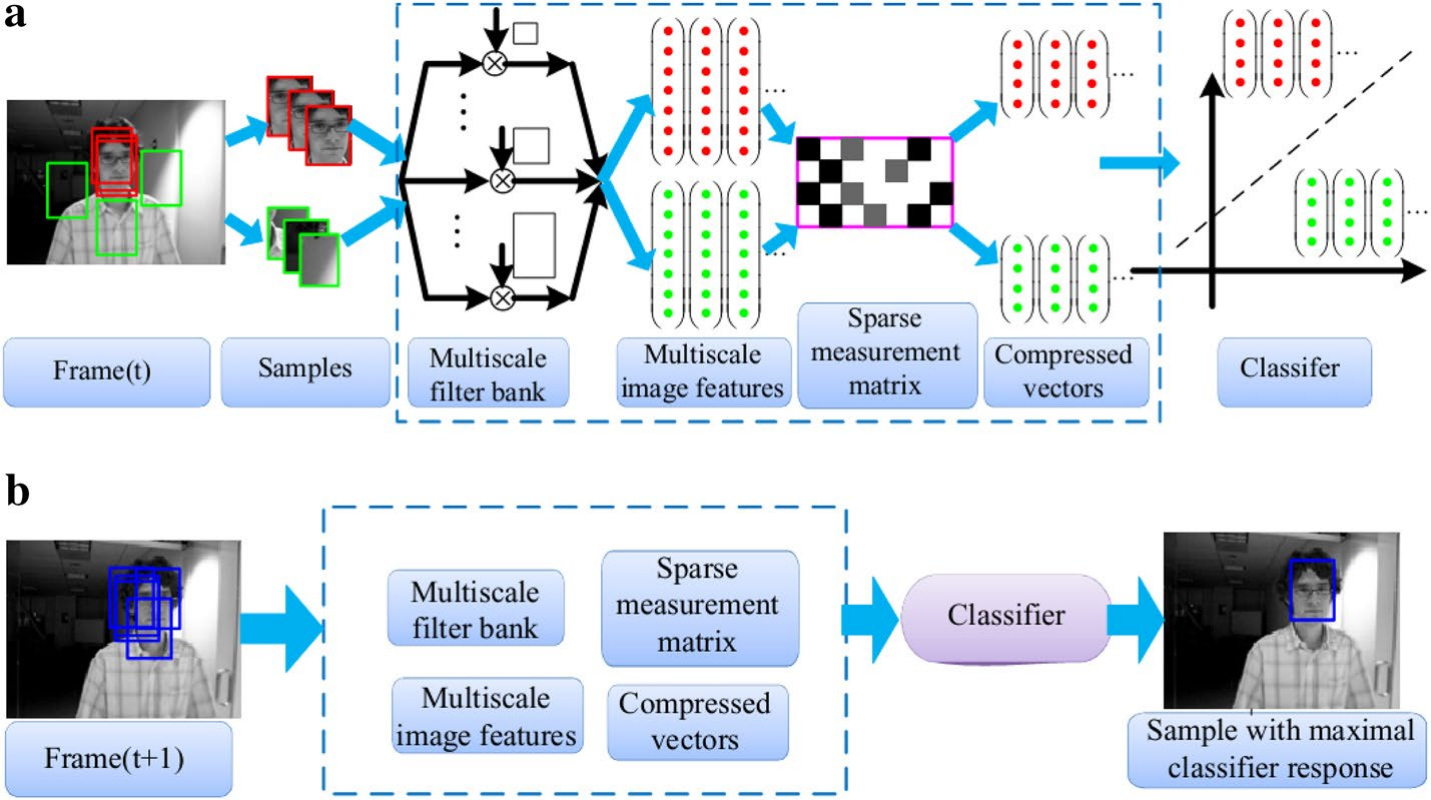
Main components of CT algorithm. **a** Updating classifier at the *t*-th frame, **b** Tracking at the frame (*t* + 1)-th

The idea of CT is motivated by the compressive sensing theory, in which the random projections of a high dimensional signal can keep the original information to a great extent (Candes and Tao 2005, 2006). The main components of CT are shown by Fig. 1. At the *t*-th frame, both positive samples and negative samples are represented by high-dimensional multi-scale vectors via convolving each patch with some rectangle filters. Then, each vector is projected onto a low- dimensional space by employing a very sparse random projection matrix that satisfies the restricted isometry property (RIP). And then, the compressed vectors are utilized to train the classifier. At the (*t* + 1)-th frame, each candidate sample is similarly processed, and then the trained classifier is utilized to search for sample with maximal classifier response. In order to analyze, we divide the CT algorithm into several steps as follows.

*Step 1*: Sample two sets of image patches $$D^{\alpha } = \{ \varvec{z}\left| {\left\| {\varvec{l}(z) - \varvec{l}_{t} } \right\| < \alpha } \right.\}$$ and $$D^{\zeta ,\beta } = \{ \varvec{z}\left| {\zeta < } \right.$$$$\left\| {\varvec{l}(z) - \varvec{l}_{t} } \right\| < \beta \}$$ with *α* < *ζ* < *β*, where $$\varvec{l}_{t}$$ is the tracking location at the *t*-th frame, *D*^*α*^ and *D*^*ζ*,*β*^ represent the positive and negative samples respectively.

*Step 2*: Each patch is transformed into a high-dimensional multi-scale vector **x** via convolving each patch with some rectangle filters at multiple scales $\{ h_{1,1} ,{ \ldots },h_{w,h} \}$ defined as

1 $$h_{i,j} (x,y) = \left\{ {\begin{array}{*{20}l} {1,} \hfill & \quad {0 < x \le i,0 < y \le j} \hfill \\ {0,} \hfill & \quad {\text{otherwise}} \hfill \\ \end{array} } \right.,$$

where *i* and *j* are the width and height of a rectangle filter, respectively. Each vector ${\mathbf{x}} \in R^{m}$ represents the multi-scale features of each patch.

*Step 3*: A random matrix ${\mathbf{R}} \in R^{n \times m}$ is employed to project the high-dimensional vector **x** onto a low-dimensional vector ${\mathbf{v}} \in R^{n}$ as ${\mathbf{v = Rx,}}$ where the entry of **R** is represented by

2 $$r_{{ij}} = \sqrt \rho \times \left\{ {\begin{array}{*{20}l} {1{\text{ }}} \hfill & \quad {{\text{with probablity}}\;1{\text{/}}2\rho } \hfill \\ {0{\text{ }}} \hfill & \quad {{\text{with probablity}}\;1 - 1{\text{/}}\rho } \hfill \\ { - 1} \hfill & \quad {{\text{with probablity}}\;1/2\rho } \hfill \\ \end{array} } \right.,$$

where *ρ* = 2 or 3, and Achlioptas (2003) has proved that this type of matrix in such a case satisfies the Johnson-Lindenstrauss lemma. Thus, each low-dimensional vector ${\mathbf{v}} =\{ v_{i} \} ,i \in [1,n]$ represents the compressive features of each sampled patch, and it can be efficiently computed using the integral image method.

*Step 4*: Each compressive feature’s conditional distributions in positive samples and negative samples both are assumed to be Gaussian distributed as *p*(*v*_*i*_|*y* = 1.) ∼ *N*(*μ*_*i*_^1^, *σ*_*i*_^1^), *p*(*v*_*i*_|*y* = 0.) ∼ *N*(*μ*_*i*_^0^, *σ*_*i*_^0^). And the low-dimensional vector ${\mathbf{v}} = \{ v_{i} \} ,i \in [1,n]$ is utilized to update Gaussian parameters

3 $$\begin{aligned} \mu_{i}^{1} &= \lambda \mu_{i}^{1} + (1 - \lambda )\mu^{1} \\ \sigma_{i}^{1} &= \sqrt {\lambda (\sigma_{i}^{1} )^{2} + (1 - \lambda )(\sigma^{1} )^{2} + \lambda (1 - \lambda )(\mu_{i}^{1} - \mu^{1} )^{2} } , \\ \end{aligned}$$

where $\sigma^{1} = \sqrt {1/n\sum\nolimits_{{k = 0\left| {y = 1} \right.}}^{n - 1} {(v_{i} (k) - \mu^{1} )^{2} } } ,\;\mu^{1} = 1/n\sum\nolimits_{{k = 0\left| {y = 1} \right.}}^{n - 1} {v_{i} (k)} ,\;\lambda$ is a constant learning parameter, its value depends on particular situations. When the object appearance change significantly, λ takes smaller value; otherwise, λ takes bigger value.

**Fig. 2:**
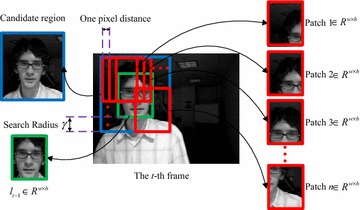
Sample patches with sliding window method

*Step 5*: Sample a set of image patches in the (*t* + 1)-th frame, $D^{\gamma } = \{ \varvec{z}\left| {\left\| {\;\varvec{l}(z) - \varvec{l}_{t} } \right\|} \right. < \gamma \} ,$ where $\varvec{l}_{t}$ is the tracking location at the *t*-th frame, and extract the features with low dimensionality. In this step, the sliding window method is used to traverse the whole candidate region to sample the patches, the sizes of which are all same to the object at *t*-th frame, illustrated in Fig. 2. It is worth mentioning that the search radius γ and one pixel distance in the figure are enlarged for show.

*Step 6*: A native Bayes classifier is utilized to distinguish the classes of each patch,

4 $$H(\varvec{v}) = \log \left( {\tfrac{{\prod\nolimits_{i = 1}^{n} {p(v_{i} \left| {y = 1} \right.)p(y = 1)} }}{{\prod\nolimits_{i = 1}^{n} {p(v_{i} \left| {y = 0} \right.)p(y = 0)} }}} \right) = \sum\limits_{i = 1}^{n} {\log \left( {\tfrac{{p(v_{i} \left| {y = 1} \right.)}}{{p(v_{i} \left| {y = 0} \right.)}}} \right)} .$$

In this step, all compressive features *v*_*i*_ in the vector **v** are assumed independent and equal contribution to the classifier (Zhang et al. 2012; Ng and Jordan 2002). By using the classifier *H*(**v**), we find the tracking location $\varvec{l}_{t + 1}$ with the maximal classifier response.

Although CT algorithm is demonstrated efficient by several experiments in Zhang et al. (2012), it has some limitations that makes CT perform unfavorably in some cases: First, in the classical CT algorithm, the estimation of scale changes of the target is not solved. Second, its constant learning parameter λ and uniform weights of Haar features are likely to bring drift when the object appearance changes significantly. In the following section, we will propose a scale adaptive CT that can deal with these issues well.

## Scale adaptive compressive tracking

### Algorithm overview

The proposed adaptive compressive tracking is summarized in Algorithm 1,
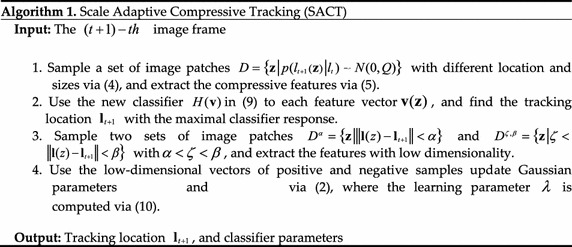
 which improved CT algorithm mainly in three aspects. Firstly, both the location and size of the object are regarded as variable parameters, which make up a vector *s*_*t*_ = (*x*_*t*_, *y*_*t*_, *w*_*t*_, *h*_*t*_). The vector is assumed to be Gaussian distributed under the assumption of Brownian motion model *p*(*s*_*t*_|*s*_*t*−1_) ∼ *N*(0, *Q*), where *Q* = *diag*(*σ*_*s*_^2^) is the covariance matrix containing diagonal elements, each corresponding to the variance of individual parameter $x_{t} ,y_{t} ,w_{t} \;{\text{and}}\;h_{t} .$ In this way, a series of patches with different size and location are sampled instead of all the patches in CT being in the same size. Secondly, the weights of the Haar features are defined by computing each feature’s ability of discriminating the object from background. These different weights are used in the classifier model instead of all the Haar features in CT having the same weight. Finally, a novel performance metric is applied to distinguish whether the current frame is reliable and low possibility of occlusion from the background or intersection from other objects. Only when the metric is satisfied, are the parameters (*μ*_*i*_^1^, *σ*_*i*_^1^, *μ*_*i*_^0^, *σ*_*i*_^0^) incrementally updated. But the parameters in CT are updated at every frame instead.

### Multi scale patches sampling and their features extraction

As illustrated by Fig. 2, the patches in CT are sampled by using sliding window method to traverse the whole candidate region. In this way, the sizes of all the patches are all same to the object at *t*-th frame. However, if the size of the object changes significantly in tracking, the drift problem is likely to occur. To handle this problem, a multi scale patches sampling method is proposed in this section, and the integral image method is still utilized to compute the compressive features efficiently.

In our algorithm, both the location and size of the object are regarded as variable parameters, which make up a vector *l*_*t*_ = (*x*_*t*_, *y*_*t*_, *w*_*t*_, *h*_*t*_), where *x*_*t*_ and *y*_*t*_ represent the center coordinates of the object at *t*-th frame, *w*_*t*_ and *h*_*t*_ are the width and height of the object at *t*-th frame. The vector *l*_*t*_ is assumed to be Gaussian distributed under the assumption of Brownian motion model

5 $$p(l_{t + 1} \left| {l_{t} } \right.) \sim N(0,Q),$$

where *Q* = *diag*(*σ*_*l*_^2^) is the covariance matrix containing diagonal elements, each corresponding to the variance of individual parameter $x_{t} ,y_{t} ,w_{t} \;{\text{and}}\;h_{t} .$ In this way, a series of patches with different size and location are sampled instead of all the patches in CT being in the same size.

**Fig. 3:**
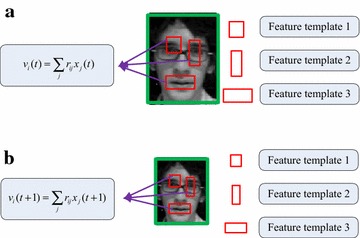
Each compressed feature is constructed by several feature templates. **a** *t*-th frame, **b** (*t* + 1)-th frame

As shown in Fig. 3, in CT algorithm, the *t*-thcompressive feature *v*_*i*_ in the compressed vector **v** is constructed by several feature templates, whose sizes and locations are set randomly and fixed during tracking. While in our proposed method, the sizes and locations of the feature templates cannot be fixed during tracking, because the sizes of sampled patches are various. The parameters of the feature templates are computed as

6 $$\begin{aligned} bx_{t + 1}^{(n)} = \frac{{bx_{t} }}{{w_{t} }} \cdot w_{t + 1}^{(n)} ,by_{t + 1}^{(n)} = \frac{{by_{t} }}{{h_{t} }} \cdot h_{t + 1}^{(n)} \hfill \\ bw_{t + 1}^{(n)} = \frac{{bw_{t} }}{{w_{t} }} \cdot w_{t + 1}^{(n)} ,bh_{t + 1}^{(n)} = \frac{{bh_{t} }}{{h_{t} }} \cdot h_{t + 1}^{(n)} \hfill \\ \end{aligned} ,$$

where (*bx*_*t*+1_^(*n*)^, *by*_*t*+1_^(*n*)^, *bw*_*t*+1_^(*n*)^, *bh*_*t*+1_^(*n*)^) represent the locations and sizes of future templates in the *n*-th sampled patches at (*t* + 1)-th frame, (*bx*_*t*_, *by*_*t*_, *bw*_*t*_, *bh*_*t*_) represent the locations and sizes of future templates at *t*-th frame, *w*_*t*+1_^(*n*)^ and *h*_*t*+1_^(*n*)^ are the width and height of the *n*-th sampled patches at (*t* + 1)-*th* frame, *w*_*t*_ and *h*_*t*_ are the width and height of the object at *t*-thframe. The integral image method is still utilized to compute each rectangular feature efficiently.

### Evaluation and application of features’ confidence

In CT, all the compressive features are supposed independent and equal contribution to the classifier (Zhang et al. 2012; Ng and Jordan 2002). Actually, different compressive features have different confidence coefficients. In our proposed algorithm, the confidence coefficients of features are computed and used to achieve different contribution to the classifier.

As the references (Abraham et al. 2013; Jing et al. 2011; Zhang et al. 2014a, b) referred to, the confidence of a feature can be represented by computing the feature’s ability of discriminating the object from background, which is computed though using the Hellinger distance between a feature’s distributions of positive and negative samples in our method

7 $$h^{2} (p(v_{i} \left| {y = 0} \right.),\;p(v_{i} \left| {y = 1} \right.)) = \frac{1}{2}\int {(\sqrt {f_{1} (x)} - \sqrt {f_{0} (x)} )}^{2} {\text{d}}x,$$

where *f*_1_(*x*) and *f*_0_(*x*) are the feature’s probability density functions (PDF) of positive samples and negative samples. Similar to CT, the distributions are assumed to be Gaussian distributed as

8 $$p(v_{i} \left| {y = 1} \right.) \sim N(\mu_{i}^{1} ,\sigma_{i}^{1} ),p(v_{i} \left| {y = 0} \right.) \sim N(\mu_{i}^{0} ,\sigma_{i}^{0} ),$$

Substituting (7) into (6), we can get

9 $$h^{2} = 1 - \sqrt {\frac{{2\sigma_{1} \sigma_{0} }}{{\sigma_{1}^{2} + \sigma_{0}^{2} }}} \exp \left( { - \frac{1}{4}\frac{{(\mu_{1} - \mu_{0} )^{2} }}{{\sigma_{1}^{2} + \sigma_{0}^{2} }}} \right).$$

It is obvious that *h* satisfies 0 ≤ *h* ≤ 1, and the bigger value *h* takes, the stronger ability of discriminating the object from background. And afterwards, the Hellinger distance *h* is utilized in the classifier to achieve the goal that features with stronger ability make more contribution to the classifier

10 $$H(\varvec{v}) = \sum\limits_{i = 1}^{n} {h_{i} \log \left( {\frac{{p(v_{i} \left| {y = 1} \right.)}}{{p(v_{i} \left| {y = 0} \right.)}}} \right)} .$$

### Online learning of features’ conditional distribution

After the tracking location has been found in a new frame, its positive and negative samples are used to update the Gaussian distribution parameters with introducing a learning parameter *λ* in CT, as (2) illustrated. However, CT suffers drift when the object appearance changes much due to its fixed learning rate *λ*. In our proposed method, a variable learning parameter *λ* is adopted, which can be adjusted according to the object appearance variation rate. To achieve this, $\rho = \sum\nolimits_{u} {\sqrt {q_{u}^{t} p_{u}^{t + 1} } }$ is utilized to compute the Bhattacharyya coefficient between the object being tracked and the last object at last frame (*q*_*u*_^*t*^ implies the histogram of the object at the *t*-th frame, *p*_*u*_^*t*+1^ implies the histogram of the object at the (*t* + 1)-th frame). It is obvious that *ρ* satisfies 0 ≤ *ρ* ≤ 1. And a larger *ρ* means the object appearance changes rapidly, consequently the Gaussian distribution parameters need a larger learning rate. On the contrary, a smaller learning rate is needed. However, when *ρ* < *Θ*, which means the current location of the object is not accurate or the occlusion has occur, the Gaussian distribution model stop update. In conclusion, the new learning parameter can be represented as

11 $$\left\{ {\begin{array}{*{20}l} {1,} \hfill & \quad {\rho < \varTheta } \hfill \\ {\lambda^{{\prime }} /\rho = \frac{{\lambda^{{\prime }} }}{{\sum\nolimits_{u} {\sqrt {q_{u}^{t} p_{u}^{t + 1} } } }},} \hfill & \quad {\rho \ge \varTheta } \hfill \\ \end{array} } \right.,$$

where $\lambda^{{\prime }}$ is the given constant learning parameter, *ρ*is the Bhattacharyya coefficient, *λ* is our new learning parameter, which can be adaptively adjusted according to the object appearance variation rate. Then *λ* in Eq. (3) will be instead by our new learning parameter, which is defined by (11).

## Experiments

We evaluate the proposed algorithm with 7 state-or-the-art methods on 50 challenging sequences, which are all among the CVPR2013 tracking benchmark (Wu et al. 2013). The 7 contrastive trackers are summarized in literature (Wu et al. 2013), containing the CSK method, the VTS method, the SCM tracker, the VTD tracker, the TLD tracker, the Struck method, and the CT method. The reason of choosing these 7 trackers is that all of them except CT has been demonstrated much better performance than other trackers, like OAB, Frag, DFT, for example. We also choose CT method to verify if the proposed tracker can improve it greatly. For fair comparison, we use the source or binary codes provided by the authors with tuned parameters for best performance. For our compared trackers, we either use the tuned parameters from the source codes or empirically set them for best results.

### Setup

The search radius of sampling positive samples is set to *α* = 3, where 50 positive samples are extracted. The inner and outer radiuses of sampling positive samples are set to *ζ* = 6 and *β* = 25, where 40 negative samples are extracted randomly. The dimensionality of projected space is set to *n* = 50, and the given constant learning parameter $\lambda^{{\prime }}$ is set to 0.8, and the threshold value is set to *Θ* = 0.5. The empirically determined parameters *σ*_*x*_, *σ*_*y*_, *σ*_*w*_, *σ*_*h*_ in *Q* are empirically chosen depending on the motion and attributes of the target in different videos. Table 1 lists the parameter values of some sequences in our experiments.

**Table 1:** Parameter Values used in the tests

Video	(*σ*_*x*_, *σ*_*y*_, *σ*_*w*_, *σ*_*h*_)	Video	(*σ*_*x*_, *σ*_*y*_, *σ*_*w*_, *σ*_*h*_)
Dudek	(5, 5, 0.3, 0.3)	Football	(3, 3, 0.1, 0.1)
Car scale	(3, 3, 0.5, 0.5)	Faceocc	(5, 5, 0.1, 0.1)
Fish	(1,1, 0.05, 0.05)	Basketball	(8, 8, 0.05, 0.05)
Car dark	(1,1, 0.01, 0.01)	Soccer	(3, 3, 0.2, 0.2)

### Experimental Results

**Fig. 4:**
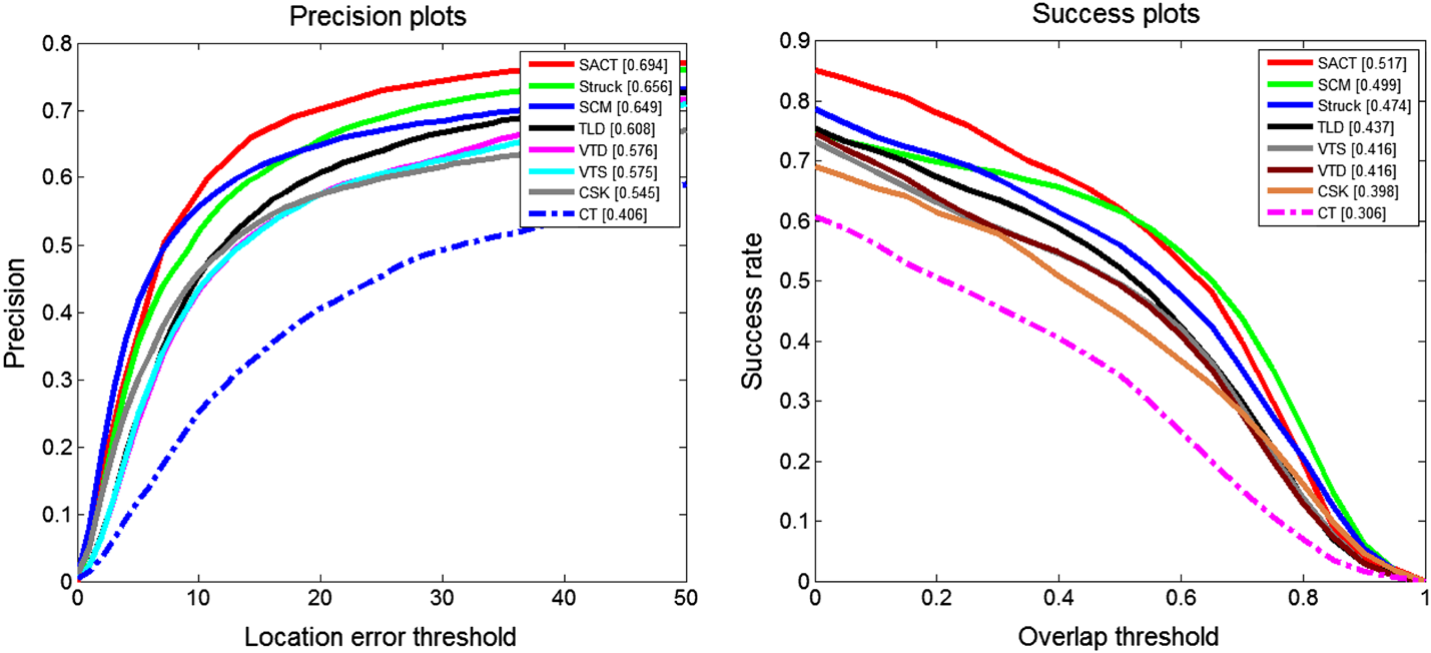
Precision plots and success plots of the 8 trackers

We use the precision plot and success plot defined in Wu et al. (2013) to evaluate the proposed algorithm with 7 state-of-the-art trackers. The precision plot shows the percentage of frames whose estimated average center location errors are within the given threshold distance to the ground truth. The score at the threshold 20 pixels is defined as the precision score. The success plot shows the percentage of frames whose overlap score are more than a threshold value, where the overlap score is defined as $SCORE = \tfrac{{area(ROT_{t} \cap ROT_{a} )}}{{area(ROT_{t} \cup ROT_{a} )}}$ with the tracking bounding box *ROT*_*t*_ and the ground truth bounding box*ROT*_*a*_. The threshold value ranges from 0 to 1, and the area under curve is used to measure the success score. Figure 4 shows the overall performance of the 7 evaluated tracking algorithms and the proposed algorithm SACT in terms of precision plot and success plot. Table 2 lists the precision score and success score for the 7 state-of-the-art trackers and SACT.

**Table 2:** Precision score and success score of the 8 trackers

	SACT	Stuck	SCM	TLD	VTD	VTS	CSK	CT
Precision plots	0.694	0.656	0.649	0.608	0.576	0.575	0.545	0.406
Success plots	0.517	0.474	0.499	0.437	0.416	0.416	0.398	0.306

**Fig. 5:**
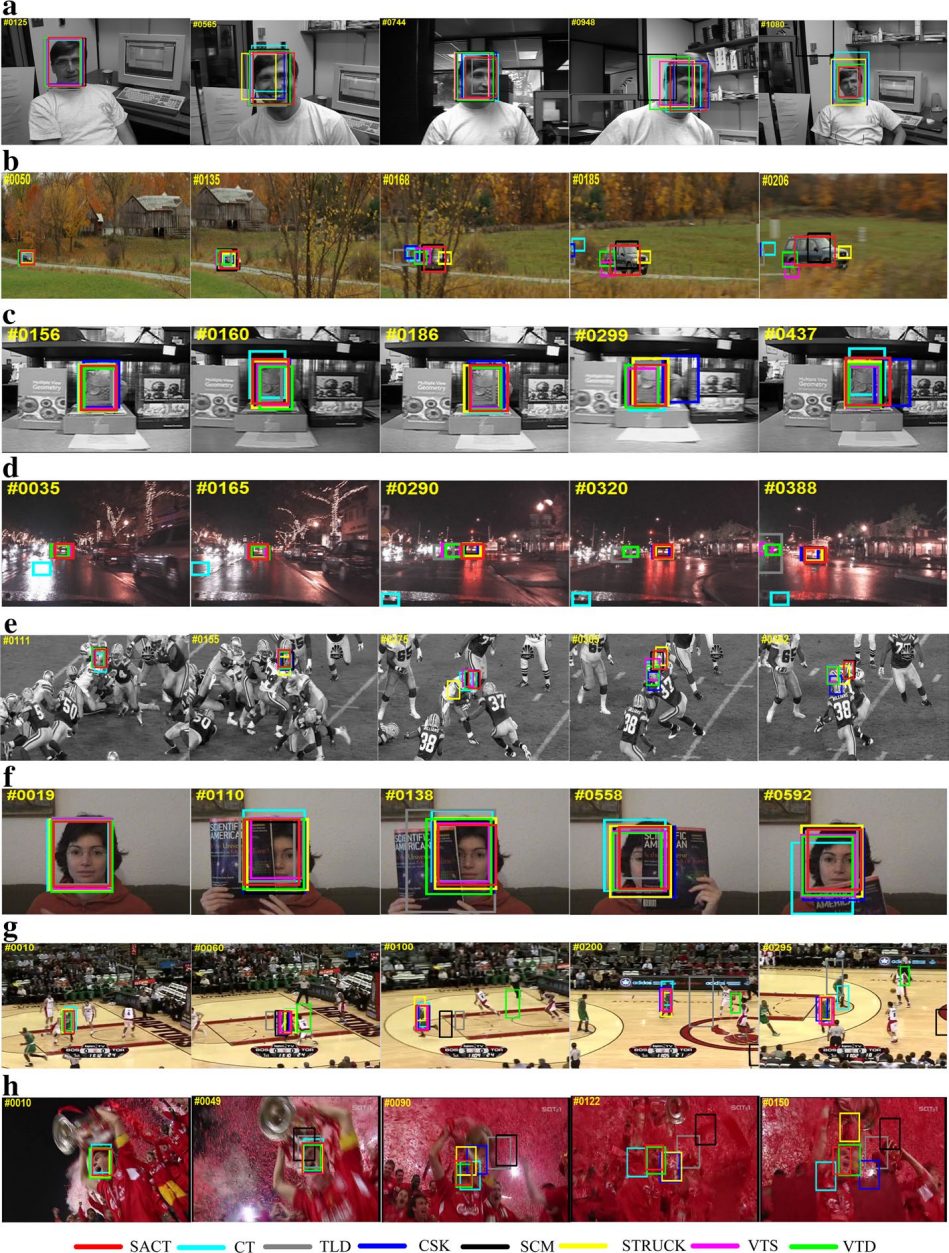
Screenshots of some sampled tracking results. **a** Dudek, **b** Car scale, **c** Fish, **d** Car dark, **e** Football, **f** Faceocc, **g** Basketball, **h** Soccer

The proposed SACT achieves the best tracking results in terms of both precision score and success score: the precision score of SACT is 0.694, which outperforms the STRUCK algorithm (ranking 2nd) by 5.79 %; meanwhile, the success score of SACT is 0.517, which outperforms the SCM algorithm (0.499 ranking 2nd). We note that the simple Haar-like features is employed to represent the object and background in the proposed algorithm SACT and the simple naive Bayesian classier with low computational complexity is adopted in SACT. Thus, the proposed algorithm SACT outperforms STRUCK and SCM that resort to complicate learning techniques in terms of both accuracy and efficiency. Besides, one can be seen from Table 2 that the proposed SACT improves CT to a large extent: the precision score of SACT outperforms 0.406 (the precision score of CT) by 70.9 %; meanwhile, the success score of SACT outperforms 0.306 (the precision score of CT) by 68.9 %. Figure 5 shows screenshots of some tracking results.

#### Scale and pose change

For the *Dudek* sequence shown in Fig. 5a, the scale and pose of the object both change gradually. The tracking results of the front half images indicate that all of these algorithms have a certain ability of dealing with pose variation (e.g., #125). But we also observe that CT, CSK and Stuck cannot deal with scale variation well due to the error caused by their constant tracking box. On contrary, other methods concluding the proposed SACT can adjust their tracking boxes according to the scale of the object (e.g., #565). Furthermore, it is obviously that the tracking box of SACT is tighter and more accurate than TLD, SCM, VTS and VTD, especially when the object gets smaller in size (e.g., #1080). The proposed SACT can deal with scale and pose variation due to the Gaussian distributed tracking box and random features selection that has been proved to handle pose variation well.

For the *Car scale* sequence shown in Fig. 5b, the object suffers from great scale change. Challenges also come from the interference caused by the tree when the object goes through it. We observed that CT, CSK and TLD drift when the object goes through the tree in the video. VTS, VTD and Struck only track a certain part of the object as it gets larger in size. On contrary, SCM and our SACT can track the object accurately in the whole sequence.

#### Illumination change

For the *Fish* sequence shown in Fig. 5c, the object undergoes several times of illumination change. The tracking result indicates that illumination getting stronger will have little effect on the tracking results of each algorithm (e.g., #156). But all the algorithms except SACT drift once the illumination get weaker (e.g., #160 and #437). The proposed SACT can deal with illumination Change in terms of its adaptive local appearance model, that is to say, different compressive features have different confidence coefficients in our tracker. For the *Car dark* sequence shown in Fig. 5d, the object undergoes large changes in environmental illumination with the car running along the street. CT, VTS, VTD and TLD drift gradually (320–388) as illumination changing while SCM, STRUCK, VTS and the proposed SACT achieve much better performance.

#### Background clutters or occlusion

The object in the *football* sequence (Fig. 5e) suffers from background clutters. Furthermore, the object also suffers from occlusion by other players, which make the sequence challenging. Overall, our tracker shows favorable performance to deal with the challenging sequence. The target in *faceocc* sequence in Fig. 5f undergoes heavy occlusion. The proposed tracker SACT achieves the best performance in terms of precision score and success score. Our tracker can handle occlusion variations and background clutters well as its adaptive appearance model and the online classifier update strategy. When the object appearance changes rapidly, a larger learning rate is applied. On the contrary, a smaller learning rate is applied. However, when*ρ* < *Θ*, which means the current location of the object is not accurate or the occlusion has occurred, the classifier stops updating. In this way, the tracker is prevented from drifting due to avoiding adding inaccurate samples.

#### Multiple challenges

The objects in the *basketball* (Fig. 5g) and *soccer* (Fig. 5h) sequences both suffer from multiple challenges, such as fast motion, motion blur, background clutters, occlusion and other challenges, which make these two sequences much challenging. Consequently, all the trackers drift to the background or other objects gradually except our tracker. Overall, SACT achieves the best performance in these two challenging sequences due to its adaptive appearance model and the online classifier update strategy.

## Conclusions

In this paper, we proposed a novel scale adaptive compressive tracking method, which improves the CT algorithm by a significantly large margin on the CVPR2013 tracking benchmark. Our method significantly improves CT in three aspects: Firstly, the scale of tracking box is adaptively adjusted according to the size of the objects. Secondly, the confidence coefficients of features are computed and used to achieve different contribution to the classifier. Finally, a variable learning parameter λ is adopted in our method, which can be adjusted according to the object appearance variation rate. Numerous experiments have shown that the superior performance of the proposed method over other 7 state-of-the-art tracking algorithms in dealing with scale and pose change, illumination change, background clutters, occlusion and multiple challenges.
